# Colorectal Liver Metastasis: Can Cytokines Make the Difference?

**DOI:** 10.3390/cancers15225359

**Published:** 2023-11-10

**Authors:** Costanza Ballarò, Valeria Quaranta, Gianluigi Giannelli

**Affiliations:** 1 Laboratory of Molecular Medicine, National Institute of Gastroenterology, IRCCS “S. de Bellis” Research Hospital, Via Turi 27, Castellana Grotte, 70013 Bari, Italy; 2 Laboratory of Personalized Medicine, National Institute of Gastroenterology, IRCCS “S. de Bellis” Research Hospital, Via Turi 27, Castellana Grotte, 70013 Bari, Italy; valeria.quaranta@irccsdebellis.it; 3 Scientific Direction, National Institute of Gastroenterology, IRCCS “S. de Bellis” Research Hospital, Via Turi 27, Castellana Grotte, 70013 Bari, Italy; gianluigi.giannelli@irccsdebellis.it

**Keywords:** colorectal cancer, liver metastasis, cytokines, chemokines, tumor microenvironment

## Abstract

**Simple Summary:**

Colorectal cancer (CRC) is one of the most common malignancies worldwide, with a mortality rate of 86%. The main cause of death is the development of colorectal liver metastases (CRLMs). The communication between cancer cells and the environment is a prerequisite for generating a favorable tumor microenvironment, and it is mediated by soluble molecules such as cytokines, growth factors, and extracellular vesicles. Mounting evidence suggests that cytokines play a decisive role in CRC progression and metastasis. In this review, we highlight the involvement of different cytokines during the development of CRLMs and emphasize the complexity as well as therapeutic implications of the cytokine milieu.

**Abstract:**

Colorectal cancer (CRC) is the third leading cause of cancer-related death worldwide. Metastasis is the prime driver of CRC-related mortality, and the liver is the organ most frequently involved. Despite the overall success of current treatments, colorectal liver metastasis (CRLM) is associated with poor prognoses and a survival rate of only 14%. Recent studies have highlighted the importance of the tumor microenvironment (TME) and the crosstalk within it in determining the invasion of distant organs by circulating cancer cells. In the TME, cellular communication is mediated via soluble molecules, among which cytokines have recently emerged as key regulators, involved in every aspect of tumor progression and the metastatic cascade. Indeed, in the serum of CRC patients elevated levels of several cytokines are associated with cancer development and progression. The current review evaluates the role of different cytokines during CRLM development. Additionally, considering the increasing amount of data concerning the importance of cytokine complex networks, we outline the potential of combination treatments using targeted cytokines together with other well-established therapies, such as immune checkpoint blockades, chemotherapy, or gene therapy, to improve therapeutic outcomes.

## 1. Introduction

Colorectal cancer (CRC) is one of the three most common malignancies and the third leading cause of cancer-related death worldwide [1]. Despite improvements in its diagnosis, screening, and treatment, the global mortality of CRC rose from 437,000 in 1990 to 915,000 million deaths in 2020 [2,3]. Additionally, the International Agency for Research on Cancer (IARC) estimates an increase from 1.9 million (data from 2020) to 3 million new cases of colon cancer by 2040 [4]. CRC metastasis remains a major problem after curative treatment and is the critical cause of CRC-related death. The liver is the organ most commonly affected by distant metastasis, mostly due to the portal vein system, which directly connects the colon–rectum and the liver [5]. Approximatively 50% of CRC patients present liver metastases at the time of the primary diagnosis or develop metastases within 5 years after the diagnosis [6]. Generally, colorectal liver metastasis (CRLM) has a poor prognosis and a survival rate of only 14–15% [7]. Surgical resection and chemotherapy are the only treatments that offer long-term survival for patients with CRLM. Nevertheless, only 10–20% of CRLM cases are suitable for resection at the time of diagnosis [7]. Currently, the direct blockading of immune checkpoints to inhibit immune escape is the most well-established immunotherapeutic approach for the treatment of several cancer types [8,9,10]. However, the response of CRC patients to immune checkpoint inhibitors (ICIs) is subject to genomic susceptibility. ICIs are more effective in patients with high DNA microsatellite instability (MSI) or deficient mismatch repair status (MMR) CRLM [11,12]. In contrast, ICIs alone or in combination show only weak outcomes in patients with microsatellite stability and/or mismatch repair proficient CRLM [13,14] Therefore, it is crucial that more effective strategies be developed both to prevent metastases spreading and to improve the survival of CRLM patients.

The tumor microenvironment (TME) is implicated in every aspect of tumor progression and metastasis, including the initial engraftment and growth. Moreover, the TME is known to be involved in regulating the response to immunotherapy, due to its ability to mediate a primary resistance or acquire resistance to cancer treatments [15]. As with most solid cancers, the CRC TME comprises not only tumor cells but also several other cell types, such as cancer-associated fibroblasts (CAFs), immune cells (e.g., macrophages, natural killer cells, dendritic cells, and T lymphocytes), and stromal cells [16]. The communication between different cell types contributes to determining the tumor fate. Cells communicate through direct cell interactions or soluble factors, such as cytokines, growth factors, extracellular vesicles (e.g., exosomes), and extracellular matrix (ECM) components. Among them, cytokines play a fundamental role in CRLM development, since their involvement in tumor initiation, invasion, and resistance to cancer treatments is well established (Table 1) [17]. Cytokines include chemokines, interferons, and interleukins, and act through cell surface receptors. Notably, it has been demonstrated that defects in cytokine signaling contribute to CRC progression and are often predictive of the worst disease evolution [18]. Recently, different studies have revealed that some cytokines enhance or inhibit the action of other cytokines in complex ways, suggesting the importance of delineating the impact of cytokine networks on carcinogenesis. In fact, due to their immunosuppressive role and, in some cases, their overlapping functions, cytokines can be considered therapeutic targets, and their inhibition may sensitize many tumors to ICIs or other immunotherapies, thus improving therapeutic outcomes. In this review we highlight how cytokine networks influence CRLM development and, therefore, the potential employment of cytokine-based therapies.

## 2. Mechanism of Colorectal Liver Metastasis Development: Different Phases, Common Cytokines

The dissemination of primary tumor cells to a specific organ is undoubtedly facilitated by anatomical factors, such as the blood flow pattern. The hepatic artery and portal venous circulation can partly explain why the liver is the preferential organ for metastatic spread, but this is not sufficient to explain the metastasis formation. Indeed, according to Steven Paget’s hypothesis, circulating cancer cells must also find a suitable microenvironment in order to colonize an organ [61]. The liver microenvironment represents a unique metastatic site for CRC circulating cells’ engraftment, survival, and growth, and several factors contribute to its composition. Among them, it is necessary remember that the liver is continuously exposed to intestinal antigens, which create an immunotolerant environment that favors CRLM [62]. Additionally, it has been shown that liver diseases such as non-alcoholic fatty liver disease (NAFLD) and alcoholic liver disease (ALD) might also improve the suitable conditions for CRLM [63,64]. Moreover, resident liver cells themselves secrete many cytokines and chemokines responsible for liver-specific colonization [65]. For example, a high expression of the chemokine CXCL12 in the liver parenchyma mediates the recruitment of CRC cells that express high levels of its receptor, CXCR4, thus contributing to hepatic metastasis [66]. Furthermore, the CCL20-CCR6 pathway plays an important role in the signaling cascade associated with the metastatic spread of CRC to the liver [67].

By analyzing genomic data, it has recently been demonstrated that the genetic difference between primary CRC cells and distant metastases in the liver and brain is small, suggesting that metastatic spread is an early event in tumorigenesis [68,69].

**Figure 1:**
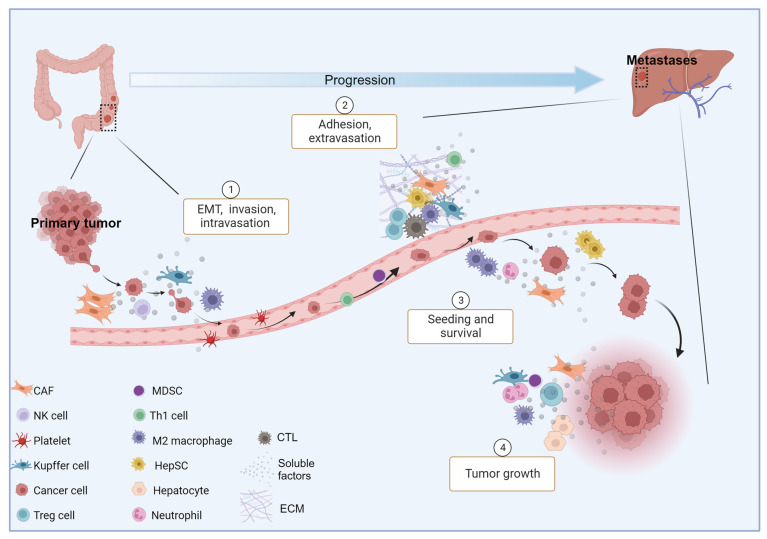
Liver metastasis cascade in CRC. Schematic representation of CRLM development through four overlapping phases: (1) microvascular, (2) extravascular, (3) angiogenic, and (4) growth. Abbreviations: CAF, cancer-associated fibroblast; CTL, cytotoxic lymphocyte; ECM, extracellular matrix; EMT, epithelial–mesenchymal transition; HepSC, hepatic stellate cell; MDSC, myeloid-derived suppressor cell; NK, natural killer; Th1, T helper 1 lymphocyte; and Treg, regulatory T lymphocyte. Created with BioRender.com.

**Figure 2:**
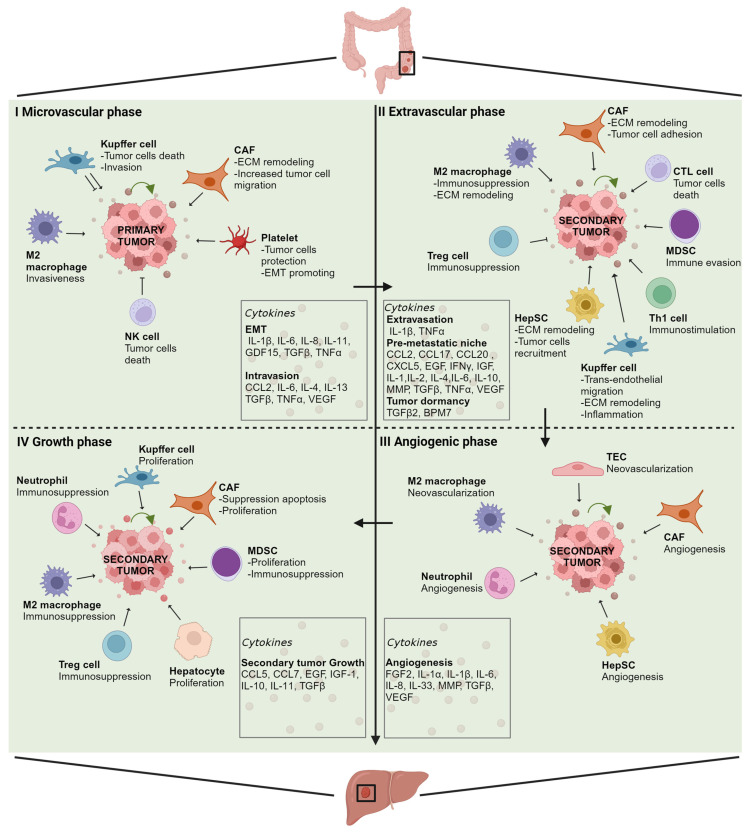
Cytokines in the pathogenesis of CRLM. Schematic representation of the cytokines involved during the different phases of CRLM development. For each phase, the contributions of the key cellular components of the CRC TME and the major cytokines involved are indicated. Abbreviations: CAF, cancer-associated fibroblast; CTL, cytotoxic lymphocyte; ECM, extracellular matrix; EMT, epithelial–mesenchymal transition; HepSC, hepatic stellate cell; MDSC, myeloid-derived suppressor cell; NK, natural killer; TEC, tumor endothelial cell; Th1, T helper 1 lymphocyte; and Treg, regulatory T lymphocyte. Created with BioRender.com.

The pathogenic development of CRLM is facilitated by two critical niches, namely the pre-metastatic niche and post-tumor invasion niche. The former is driven by factors secreted by the primary tumor that, in turn, recruit non-parenchymal cells, including Kupffer cells (KCs, resident liver macrophages), hepatic stellate cells (HepSCs, pericytes in the perisinusoidal space of the liver), myeloid-derived suppressor cells (MDSCs, immune cells from the myeloid lineage), and neutrophils. The latter, instead, develops following tumor cells’ entry into the liver, and proceeds through different phases. These include (1) a microvascular phase, when cancer cells infiltrate and become trapped in liver vessels; (2) an extravasion and pre-angiogenic phase, when invading tumor cells relocate in the liver and collaborate with stromal cells to form a supportive TME; (3) an angiogenic phase that leads to the formation of intra-metastatic vessels; and (4) a growth phase, where metastatic cells acquire an adequate blood supply and proliferate rapidly to form a detectable tumor (Figure 1). It has been demonstrated that cytokine-mediated signaling networks affect each phase of CRC progression in several ways [17] (Figure 2).

### 2.1. EMT

The epithelial–mesenchymal transition (EMT) is a process that occurs when epithelial cells lose their characteristics (cell polarity, cell–cell adhesion, and cell–matrix adhesion) and acquire a mesenchymal phenotype [70], thereby gaining migratory and invasive properties. Transcription factors such as SNAIL, TWIST, and ZEB repress the expression of adhesion molecule E-cadherin in favor of increased levels of the mesenchymal adhesion molecule N-cadherin, thus suppressing the cancer cells’ epithelial phenotype and activating the mesenchymal phenotype. Intriguingly, it has been demonstrated that in CRC cells the expression of these transcription factors is often upregulated [71,72].

Different cytokines play a fundamental role in the EMT. Tumor necrosis factor α (TNFα) is a key inflammatory cytokine produced by both hematopoietic and non-hematopoietic cells. Intestinal epithelial cells are highly sensitive to TNFα due to high levels of expression of its receptor, TNFR1, which potently activates NF-kB oncogenic pathways [51,52]. TNFα is involved in the EMT through increasing the activity of SNAI1, an effect mediated by AKT-dependent GSK-3β inactivation [73]. Finally, TNFα is also involved in CRC cells’ migration through upregulating TROP-2, a transmembrane glycoprotein strongly associated with tumor invasion and metastasis [74]. Interleukin-1β (IL-1β) is a cytokine expressed at high levels in several cancer types, and its expression increases during the progression of CRC. IL-1β has been found to promote the EMT phenotype in colon cancer cells through ZEB1 upregulation, since Zeb1 knockdown cells display the reversal of the IL-1β-induced EMT [26]. Moreover, neutralization experiments using anti-IL-1β-specific antibodies showed that IL-1β is sufficient to activate Wnt signaling through the inhibition of GSK-3β, thereby inducing mesenchymal characteristics [27].

IL-6 is a crucial mediator of inflammation and immunity produced by diverse cell types (e.g., T cells, macrophages, and CRC cells). It has several important roles in CRC progression, including triggering the EMT via JAK2/STAT3 signaling [31]. STAT3 activation upregulates CEACAM 5, which plays a pivotal role in cell adhesion, migration, and metastasis in CRC [75]. Recent studies have demonstrated that NOTCH signaling contributes to CRC pathogenesis due to its role in the EMT induction [76]. Intriguingly, supernatants from human colon cancer mesenchymal stem cells enhance the invasive activity of CRC cells in vitro [77]. Notably, IL-6 is induced by various factors, such as TNFα and NF-kB [53]. IL-11, a member of the IL-6 family, is highly expressed in human CRC and is also a stronger STAT3-activating cytokine than IL-6 [43]. Genetic variation in the genes encoding IL-8 and its receptor CXCR2 has been unveiled as another important modulator of tumorigenesis, being associated with CRC progression, therapeutic resistance, and tumor recurrence [37]. Accordingly, the transgenic expression of IL-8 in mouse skin promotes the outgrowth and metastasis of subcutaneous CRC xenografts [38]. Conversely, the EMT- inducing transcription factor SNAI1 can induce IL-8 expression in CD44+ cancer stem-like cells, and both are co-expressed in human CRC [78]. Recently, GDF15, a member of the transforming growth factor β (TGFβ) superfamily, has been characterized as a biomarker for screening early stage CRC, and its expression is correlated with liver metastasis and poor survival [79]. Consistent with this, an expression analysis performed on human gastric tumor tissues showed a high expression of GDF15 in gastric cancer cells, correlated with invasive potential. Moreover, the overexpression of GDF15 in a human gastric cell line increases both the activity of urokinase-type plasminogen activator and the expression of its receptor, parameters associated with tumor invasiveness. TGFβ is a multifaced cytokine that exhibits a double role in CRC. In early stage cancer it acts as a tumor suppressor by inducing cell cycle arrest and apoptosis, while in late-stage cancer it promotes tumorigenesis, being the central activator of the EMT [80]. TGFβ induces the SMAD pathway (the canonical pathway) and non-SMAD signaling pathways (non-canonical pathways, such as MAPK and PI3K) to control multiple processes in carcinogenesis [81]. For example, TGFβ can induce the secretion of IL-11 by CAFs, which triggers STAT3 signaling in CRC and confers a high metastatic capacity to them [44]. In addition, it has been shown that TNFα accelerates the TGFβ-induced EMT, and this process depends on enhanced p38 MAPK activity [54]. FLH2 is an important adaptor and modifier in protein interactions, and is highly expressed in primary and metastatic colon cancer cells [49]. It acts as a potent inducer of the EMT by stimulating vimentin as well as matrix metalloproteinases-9 (MMP-9) and inhibiting E-cadherin expression. Nevertheless, it has been demonstrated that FHL2 expression is stimulated by TGFβ in a SMAD-independent manner [49]. An in vivo study reported that elevated levels of TGFβ in adjacent stromal cells increase liver metastasis formation by CRC cells [44], while the inhibition of stromal TGFβ signaling was found to block liver metastasis initiation in vivo and suppress angiogenesis in vitro [82,83]. All of this evidence suggests that TGFβ provides a continuous stimulus that promotes metastasis in CRC.

### 2.2. Microvascular Phase

When CRC cells undergo the EMT they can invade surrounding tissues, intravasate into the blood, and pass through the portal venules into the liver capillaries, called sinusoids. Although the process of intravasation is still poorly understood, it is known that to facilitate circulating CRC cells’ invasion of the underlying tissue the initial steps require the disruption of the endothelial cell–cell junctions and the basement membrane, both processes mediated by MMPs secreted by CAFs, tumor cells, and tumor-associated macrophages (TAMs) [84]. TAMs are conventionally categorized as two subtypes, namely M1 and M2. M1 macrophages are mainly involved in proinflammatory responses and have antitumor effects, while the M2 subtype has anti-inflammatory effects and promotes tumor growth. In particular, M2 macrophages are activated by several cytokines and are implicated in different protumoral activities, such as immunosuppression, invasiveness, and angiogenesis [32]. Interestingly, using a zebrafish model, it has been demonstrated that IL-6 or TNFα-activated M2 macrophages can physically interact with CRC cells, thus facilitating cancer cells’ intravasation and thus promoting tumor metastasis [32]. Tumor cells’ intravasation in the sinusoids triggers a rapid answer by the liver, which adopts several defense mechanisms in order to protect the organ and thereby the entire organism against disseminating CRC cells. KCs represent the first line of defense, since they can kill tumor cells via phagocytosis and by releasing proteinases as well as TNFs, both processes potentiated by natural killer (NK) cells [85]. The death of tumor cells and tissue damage lead to the release of various inflammatory interleukins, including IL-1, IL-6, IL-8, and IL-18, as well as chemokines such as CCL5 [39]. In turn, these cytokines can recruit more immune cells to enhance local tumor immunity. On the other hand, this local inflammatory response can also lead to the progression to the extravascular phase by upregulating the expression of adhesion molecules necessary for the extravasation [19]. Finally, a recent study demonstrated that KCs also have a prometastatic role. Indeed, KCs could be recruited by the TCF4-CCL2/CCR2 pathway, which is overexpressed in CRC, and be polarized into the M2 subtype, which can secrete protumor cytokines such as IL-4, IL-13, vascular endothelial growth factor (VEGF), and epidermal growth factor (EGF) [25]. Furthermore, it has been shown that KCs and HepSCs are involved in immune surveillance escape through two mechanisms: by releasing the chemokine CXCL1, and thus inducing MDSC recruitment, and by secreting CCL5, thus inhibiting cytotoxic T cells’ function [17,85]. One way to circumvent death via phagocytosis derives from platelets: it has been demonstrated that platelets coat tumor cells to form a physical barrier that protects them against an immunological attack [25]. Furthermore, after binding CRC circulating cells, platelets secrete TGFβ and platelet-derived growth factor (PDGF), which maintain the EMT state of tumor cells and contribute to the immunosuppression of NK cells [86].

### 2.3. Extravascular and Pre-Angiogenic Phase

The fraction of CRC circulating cells that escape immune surveillance must adhere to the endothelium of sinusoids to extravasate and form a metastatic lesion. The liver sinusoidal endothelial cells (LSECs) form the lining of the hepatic sinusoids and they control the trafficking of molecules as well as cells between the blood and the liver. They express E-selectin, a cell adhesion molecule, itself induced by cytokines and involved in CRC cell adhesion to LSECs [87]. Under physiological conditions, LSECs express low levels of E-selectin. However, Khatib et al. showed that once CRC circulating cells intravasate, they activate KCs to produce TNFα and IL-1β. In turn, these cytokines induce the expression on LSECs of E-selectin and other adhesion molecules, such as vascular cell adhesion molecule-1 (VCAM-1) and intercellular adhesion molecule-1 (ICAM-1) [19]. These adhesion receptors mediate CRC cell adhesion onto endothelial cells, thus promoting trans-endothelial migration and, therefore, successful extravasation into the liver parenchyma [88].

#### 2.3.1. Preparation of a Pre-Metastatic Niche

The term “pre-metastatic niche” refers to the microenvironment in a secondary organ that provides favorable conditions for colonization by tumor cells. It is required for CRLM development because, in the liver, the arriving tumor cells find immune cells, parenchymal and non-parenchymal liver cells, all contributing to create a hostile environment for the survival of cancer cells. Thus, the preparation of a pre-metastatic niche is an event occurring prior to the arrival of CRC cells, mandatory to escape immune surveillance and contemporarily transform the local stroma. The first contribution is made by the primary tumor itself, which prepares the distant niche by releasing secreted factors that, in turn, recruit KCs, HepSCc, MDSCs, and neutrophils [5]. In particular, HepSC activation plays a crucial role in the liver TME remodeling by secreting TGFβ, EGF, VEGF, insulin-like growth factor (IGF), and MMPs [50,89]. CRC cells produce VEGF-A, which stimulates the expression of the chemokine CXCL1 by TAMs in a primary tumor. Elevated levels of CXCL1 recruit CXCR2-positive MDSCs to pre-metastatic liver tissue and promote host immune response evasion, thus increasing tumor cell survival [23]. To achieve this goal, MDSCs attenuate the immunoreaction of NK cells by releasing nitric oxide, which interferes with NK cell functions [90]. Recently, it has been shown that STAT3 signaling in CRC cells results in the production of IL-6, which, in turn, is involved in the recruitment of MDSCs [33]. Studies have indicated that NOTCH1 signaling in a tumor induces TGFβ2 expression in the hepatic pre-metastatic niche, resulting in TGFβR1-dependent neutrophil recruitment to the liver. In turn, neutrophils inhibit cytotoxic T lymphocytes (CTLs, cells involved in the elimination of mutated and cancerous cells), thereby contributing to immunosuppression in pre-metastatic niches [91]. Further immunosuppression can be achieved through the recruitment of MDSCs by serum amyloid A1 and A2 (SSA1 and SSA2) proteins, released from parenchymal hepatocytes after IL-6-mediated STAT3 signaling activation [92]. CD4+ T lymphocytes include T helper 1 (Th1), T helper 2 (Th2), and regulatory T (Treg) cells. Th1 and Th2 cells are involved in the antitumor immune responses. In particular, Th1 lymphocytes stimulate macrophages to phagocytose, thus aiding cell-mediated killing. They exert their function by producing cytokines such as interferon γ (IFNγ) and TNFα. Instead, Th2 lymphocytes are involved in the activation of humoral immunity by secreting IL-4 [24]. Finally, Treg lymphocytes act to suppress immune responses, thus facilitating cancer immune evasion [21]. The immunosuppressive mechanism of action of Treg cells occurs partly by involving cytokines: Treg cells sequester high amounts of IL-2, thus lowering its availability for activating CTLs. On the other hand, Treg cells are able to inhibit the activation of CTLs by releasing IL-10, IL-35, and TGFβ [41,93]. In this context, TGFβ has been recognized not only as a strong inducer of the polarization of intratumor Tregs, but also as an important player in regulating Tregs’ motility across the endothelial layer, thus facilitating their recruitment at the tumor site [94]. The exposition to continuous cancer antigenic stimulation leads to the transformation of CD8+ T cells into a dysfunctional state, known as “exhaustion” [25]. These exhausted cells are impaired in promoting tumor-killing effects due to the loss of the effector function, that is, the ability to secrete effective cytokines, including IL-2, IFNγ, and TNFα [25]. Via CCL2/CCR2 signaling, CRC cells contribute to the formation of a favorable TME by controlling the recruitment of M2 macrophages [20]. In turn, M2 cells secrete cytokines such as CCL22, CCL17, IL-10, and TGFβ, which support an immunosuppressive microenvironment [42]. In addition, M2 macrophages contribute to cancer invasion by producing MMPs, which lead to matrix remodeling and thus facilitate metastasis [34]. Furthermore, it was demonstrated that IL-1β also contributes to cell invasion through the activation of protein kinases AP-1 and NF-kB by stimulating the overexpression of MMP-2 and MMP-9 [29]. The dendritic cells (DCs) in the liver inhibit T lymphocytes’ function by secreting high levels of IL-1 but low levels of IL-12, thus maintaining liver tolerance [45]. Tumor-associated neutrophils (TANs) are another immune cell type that contribute to the immunosuppression of the hepatic microenvironment. Indeed, TANs recruit M2 macrophages and Treg cells by producing CCL2 and CCL17. In turn, M2 and Treg cells favor the establishment of an immunosuppressive TME in the liver, thus promoting cancer progression and metastasis [95].

Several studies have highlighted a mechanism in which CRC-derived exosomes, which contain different kinds of bioactive molecules, promote the transformation of the liver TME [96]. For example, pancreatic cancer-derived exosomes contain a large amount of migration inhibitory factor (MIF), a cytokine which is taken up by KCs through fusions with exosomes. KCs respond by producing TGFβ, leading to the activation of HepSCs, and the subsequent deposition of fibronectin [56]. The blockade of MIF activity prevents liver metastases, thus confirming the importance of cytokines in mediating the adhesion of circulating tumor cells to distant sites in the metastatic cascade. Other well-known components of tumor cell-derived exosomes released by CRC cells are miRNAs, which also play a role in the pre-metastatic niche preparation. Exosomal miR-21-5p activates the polarization of KCs to the M2 phenotype, which in turn synthesizes IL-6, thereby creating an inflammatory pre-metastatic environment [97]. In a recent study, Zhao et al. found that the exosome cargo miR-181a-5p activates HepSCs by targeting SOCS3 and activating the IL-6/STAT3 signaling pathway. In turn, activated HepSCs remodel the liver microenvironment via the activation of the CCL20/CCR6 axis in CRC cells [22]. Tan et al. demonstrated that the interaction between CRC cells and HepSCs could promote the differentiation of HepSCs into CAFs via the TGF-β/CXCR4 axis [98]. Among the tumor-derived exosome cargo components there is also TGFβ, which is involved in the transformation of normal quiescent fibroblasts into CAFs, which modify the TME, as discussed above [57]. Finally, a recent study suggests that CRC-derived exosomes containing HSPC111, a ribosome component, reprogrammed the lipid metabolism in HepSCs, resulting in increased CXCL5 secretion that recruited tumor cells through the CXCL5-CXCR2 pathway [99].

#### 2.3.2. Tumor Cell Dormancy

After the invasion of the liver pre-metastatic niche, tumor cells may enter a quiescent state called “tumor dormancy”. During this phase, CRC dormant cells enter the G0 phase of the cell cycle and express weak tumor antigens, thereby escaping immune surveillance, surviving and adapting in the changing microenvironment. Moreover, tumor dormant cells may go undetected for long periods, thus explaining the absence of clinical symptoms, until they awake as aggressive metastases. Several cytokines and signaling pathways have been proposed to be regulatory factors of tumor dormancy. Among them, the TGF family members TGFβ2 and bone morphogenetic protein 7 (BPM7) may activate p38 MAPK, which results in the inhibition of p21 and p27, leading to cell cycle arrest and cellular quiescence [58,100].

### 2.4. Angiogenic Phase

A metastatic vasculature is established through VEGF-dependent tumor angiogenesis. This process is essential for providing the oxygen and nutrients necessary for the growth of the primary tumor and metastases. Alternatively, existing blood vessels can be co-opted independently of VEGF, thus explaining why the majority of patients do not exhibit a response to an anti-VEGF agent in addition to chemotherapy [59]. Moreover, other molecules, such as fibroblast growth factor 2 (FGF2), secreted by neutrophils recruited to the liver, can promote the growth of metastasis and vascular remodeling in the liver [101]. HepSCs and CAFs are also involved in this phase because they produce proangiogenic factors such as VEGF, angiopoietin-1, and IL-8 [38,60]. In contrast, a reduction in CXCR2 diminishes tumor angiogenesis and enhances tumor necrosis [102]. CRC-derived IL-1α augments angiogenesis by modulating stromal cells within the TME and facilitates metastasis [103]. IL-17F may also be implicated, since its overexpression in HCT116 CRC cells inhibits angiogenesis and reduces tumor growth [104]. While endothelial cells (ECs) present in the innermost layer of blood vessels promote selective permeable exchange between the blood and tissue, tumor-associated ECs (TECs) do not form a regular single layer and do not have a normal barrier function [105]. TECs promote neovascularization and CRLM by secreting IL-33 [46]. TGFβ1, also secreted by CRC cells during this phase, exerts its prometastatic function by affecting the crosstalk between the tumor and TECs, thereby promoting angiogenesis. Thus, TGFβ1 silencing significantly reduces liver metastasis formation in vivo and suppresses angiogenesis in vitro [82]. IL-6 has a profound effect on enhancing angiogenesis through the activation of STAT3 signaling [35]. Finally, it has been demonstrated that IL-1β can also promote colon tumor growth, inducing angiogenesis [106].

### 2.5. Growth Phase

After blood vessels’ formation or co-option, metastatic tumor success depends on further tumor growth. A rigorous remodeling of local stroma is necessary and is accompanied by metabolic adaptations of disseminated CRC cells in order to allow them to grow out into macroscopic metastases. Signals from the liver microenvironment stimulate factors to escape dormancy and induce tumor growth. For example, the chemokine CCL7, secreted by MDSCs, binds to CCR2 on disseminated CRC cells, thereby activating the STAT3 signaling pathway, resulting in growth and colonization [44]. Similarly, GP130/STAT signaling induced by IL-11 derived from TGFβ-stimulated CAFs can suppress apoptosis and promote outgrowth to CRLM [107].

## 3. Cytokine Network and Therapy for CRLM

Cytokines have been known for their antitumoral efficacy since 1976, when a preclinical study demonstrated that treatment with human IFN had an antileukemic effect [108]. Since then, many cytokines have been investigated as potential therapeutic agents or targets. For example, a statistical analysis showed that CRC patients with low TNFα serum levels had a significantly higher survival rate compared to patients with high levels of TNFα [109]. Accordingly, a preclinical study showed that, in a CRC mouse model, the administration of Etanercept, a specific antagonist of TNFα, strongly decreased tumor progression [110]. Another report stated that blocking TNFα signaling reduces the number of liver metastases induced by human colorectal carcinoma CX-1 cells [111]. Moreover, a phase I trial has determined the side effects and best dose of NGR-hTNF, a fusion protein that delivers TNFα to tumors, in order to induce an antivascular effect (NCT00098943) [36]. In CRC, liver metastases are supported by CAFs, which are recruited to produce a prometastatic microenvironment through the activation of inflammatory IL-6 [36]. The genetic ablation of IL-6 reduces tumor development in colitis-associated colorectal cancer (CAC) [112]. Nowadays, a wide range of therapeutic approaches has been developed to target the IL-6/STAT3 pathway in the treatment of CRC [113]. IL-2 exerts its antitumor effect by stimulating T cell proliferation [21]; therefore, it is considered a potential drug candidate. This is reflected by a high number of clinical trials targeting IL-2. Among them, a phase I/II study is currently evaluating the safety and pharmacodynamics of MDNA11, a recombinant IL-2 engineered to preferentially activate immune effector cells (CD8+ T and NK cells) in patients with advanced solid tumors (NCT05086692). Many studies have reported that IL-1β plays a pivotal role in tumor growth and metastasis [114,115]. Indeed, the knockout of the IL-1 receptor (IL-1R) in the intestinal epithelium alleviated tumor formation and progression in mouse models of CRC [116]. Inhibitors at different levels of IL-1β synthesis and signaling pathways help in CRC therapy [117]. In addition, the human IL-1R antagonist Anakinra inhibits both IL-1 and its receptor, IL-1R, reducing metastatic CRC [118]. Recently, a phase III trial (NCT02138422) has revealed that Xilonix, a monoclonal antibody against IL-1alpha, not only slows tumor growth, but also improves symptoms of muscle loss, fatigue, and appetite loss in patients with CRC [47]. Emerging studies indicate that the IL-33/ST2 axis may be a new therapeutic target in CRC. Consistently, increased levels of IL-33 and increased IL-33+ microvessel densities occur in the stroma of adenomas and CRC. In support of this, the administration of the soluble form of the IL-33 receptor suppresses tumor growth by inhibiting IL-33-induced angiogenesis [48]. The CXCL12-CXCR4/CXCR7 axis is associated with recurrence and metastases in CRC [119]. Multiple antagonists of CXCR4 are currently tested in preclinical and clinical studies on various cancers. In particular, the CXCR4 inhibitor LY2510924 has been proven to be clinically safe in CRC and pancreatic cancers, with the primary response being 20% (NCT02737072) [120]. Nevertheless, targeting CXCR4 or CXCR7 alone is not sufficient for inhibiting tumor progression, suggesting that other pathways are activated and that both receptors need to be blocked [40]. More recently, Spiegelmers have been used as an alternative approach to inhibit the CXCL12-CXCR4/CXCR7 axis. These are synthetic oligonucleotides conjugated to polyethylene glycol to target CXCL12 and prevent binding to its receptors [121]. Currently, there is an ongoing phase II study on the Spiegelmer Olaptesed pegol combined with Pembrolizumab and nanoliposomal Irinotecan/5-FU/Leucovorin or Gemcitabine/Nab-paclitaxel in microsatellite-stable metastatic pancreatic and colorectal cancer patients (NCT04901741). TGFβ is a potent activator of the EMT, invasion, and metastasis by controlling different genes and proteins related to cell–cell adhesion and ECM remodeling [89]. Several classes of TGFβ-based therapies are currently undergoing clinical trials (https://clinicaltrials.gov/, accessed 4 September 2023): monoclonal neutralizing antibodies against TGFβ ligands, antisense oligonucleotides, small-molecule inhibitors, and gene therapy. For instance, an antisense oligonucleotide specific to the mRNA of TGFβ has been positively tested in a phase I trial on various solid cancers (NCT00844064). Additional studies are needed, for example, to determine whether an anti-TGFβRII monoclonal antibody (IMC-TR1) could be safe and tolerable in participants with solid tumors, including in CRC (NCT01646203). Moreover, the oncogenic and tumor-suppressive role of TGFβ, as well as the complexity of its function in the modulation of cellular and tissue homeostasis, often lead to unsatisfactory therapy outcomes [30]. IL-11 is highly expressed in human CRC and its receptor blockade can attenuate CRC xenograft growth. Based on this study and reports that TGFβ promotes metastasis by inducing IL-11 production by CAFs, IL-11 has emerged as another possible target to use in cancer therapy [43]. In addition, a randomized phase II trial has demonstrated that IL-11 is also effective for the treatment of chemotherapy-induced thrombocytopenia in patients with recurrent colorectal cancer (NCT03823079). Genetic variations in the genes encoding IL-8 and its receptor, CXCR2, are associated with CRC progression, therapeutic resistance, and tumor recurrence [37]. Indeed, antagonism of CXCR2 with the small molecule SCH-527123 impairs cell proliferation, motility, and invasiveness in CRC xenografts [122]. As of now, there are 29 distinct clinical trials targeting CXCR2; among CXCR2 antagonists, SX-682 is being tested in different malignancies such as melanoma, myelodysplastic syndromes, and pancreatic cancer. Further studies are required to test its efficacy in CRLM.

Cytokines have two main peculiarities: redundancy and pleiotropy. While the former means that different molecules exert the same effect, pleiotropy allows the same proteins to exert different effects. Therefore, cytokines are key players in carcinogenesis as they may exert pro- and antitumoral effects, which might also co-exist in different phases of the disease. Recent studies have focused more on targeting cytokine signaling to improve anticancer responses in CRLM. For example, IL-4 is involved in promoting the EMT in CRC, and it has been reported to protect CRC cells from apoptosis. Interestingly, the inhibition of IL-4 signaling blocks resistance to chemo-immunotherapeutic treatments [123]. On the other hand, complex cytokine interactions make it difficult to identify effective targets for therapeutic strategies. For example, IL-17 is an enhancer of CRC progression, and it has been shown that IL-17 and TNF synergize to promote carcinogenesis through the high production of growth factors by CRC cells [124]. IL-22 is another promoter factor of IL-17 activity. Indeed, although inhibiting IL-17 reduces intestinal inflammation, only IL-22 neutralization has been shown to significantly reduce the dysplasia and tumor burden in a mouse model of CRC [125]. Tumor-infiltrating leukocytes (TILs) secrete multiple cytokines that promote the proliferation of CRC cells both through STAT3 (induced by IL-6 and IL-22) and NF-kB (induced by TNFα and IL-17A) signaling pathways. Consistently, in vivo findings demonstrated that the proliferative stimulus could not be abolished through the inhibition of individual cytokines. Only the combinatorial blockade of IL-6, IL-22, TNFα, and IL-17A could effectively neutralize the mitogenic effect induced by TIL-secreted cytokines [53].

Cytokine families could also be broadly inhibited by targeting shared receptor subunits. For example, the blockading of GP130 can inhibit the signaling of several cytokines, including IL-6, IL-11, and leukemia inhibiting factor (LIF) [126]. Similarly, the blockading of IL-22RA1 can impair not only IL-22-induced signaling but also that of IL-20 and IL-24 [127]. Therapy with bispecific antibodies to target distinct cytokine pathways using a single therapeutic agent is an intriguing option for CRLM [128]. For example, the use of a novel anti-TGFβ/VEGFA bispecific antibody, Y332D, entirely counteracts the in vitro biological functions of TGFβ and VEGFA, including the EMT and immunosuppression. Consistently, the in vivo experiment data demonstrate that Y332D exerts a superior antitumor effect compared to anti-TGFβ and anti-VEGF monotherapies [129]. Similarly, several clinical trials have recently investigated the antitumor activity of a modified IL-2 in combination with both of the EGFR inhibitors Trastuzamab and Cetuximab (NCT02627274) and a monoclonal antibody targeting VEGF (NCT03063762).

To summarize the above findings, the multitude of protumorigenic cytokines involved in CRLM development implies that cytokine-based therapies may be advantageous; however, other considerations need to be made. As cytokines’ roles range from immune regulation to antimicrobial activity and responses to inflammation, altering cytokines’ signaling may significantly impair host immune homeostasis. In addition, conventional treatments that target cytokines are sometimes accompanied by toxic side effects such as headaches, fatigue, and injection site reactions. Thus, how can the side effects of cytokine-based therapy be reduced? A better approach would be to use a specific vehicle to precisely target selected cytokine signaling.

## 4. Conclusions

In this review, we summarized data indicating that cytokines promote several key hallmarks of cancer, including aberrant growth, angiogenesis, invasiveness, and metastasis. Preclinical and clinical research has led to the therapeutic use of cytokines in tumor therapy, and, more recently, in preventing CRC invasion as well as liver metastasis. Despite the overall success of current treatments, the prognosis of patients with liver metastases remains poor. Blocking protumorigenic elements and simultaneously promoting antitumor immunity may improve the efficacy of therapies; therefore, combining the manipulation of cytokine pathways with checkpoint blockading can represent a promising strategy. The current knowledge of cytokine biology and immunotherapy has led to an increase in the number of ongoing clinical trials involving more than one strategy; however, multitarget approaches have both positive and side effects, including systemic proinflammatory effects due to the use of cytokines. One approach could be targeting agents directly to the TME in order to neutralize the immunosuppressive function of cytokines. On the other hand, in this age of the expansion of precision and personalized medicine, it would be useful to develop novel prognostic biomarkers. Recent studies point to cytokines as potential biomarkers for diagnosis and monitoring the clinical outcomes of CRLM patients. The assays with which to detect cytokines still have a sensitivity limit due to the low concentration of cytokines, their thermal instability, and other factors. Therefore, future studies are needed to confirm the consistency of the results and their applicability.

## Figures and Tables

**Table 1:** Main cytokines involved in CRLM development.

Cytokine	Cellular Source(s)	Effects on CRLM	Reference
CCL2	Cancer cells	Promotes immune cell recruitment, inflammation, and angiogenesis	[19,20]
CCL20	Cancer cells	Promotes the recruitment of Tregs and Th2 cells, metastasis, and TME remodeling	[21,22]
CXCL1	TAMs	Promotes immune response	[23]
IFNγ	Th1 cells and TECs	Promotes angiogenesis, inflammation, and immune responses	[24,25]
IL-1 β	TAMs, CAFs, neutrophils, and KCs	Promotes cancer cell proliferation, the EMT, and TME remodeling	[26,27,28,29]
IL-4	Th2 cells	Promotes inflammation and immune responses	[19,24,30]
IL-6	M1 macrophages, neutrophils, TECs, CAFs, and cancer cells	Promotes inflammation, migration, angiogenesis, cancer cell proliferation, and immune responses	[31,32,33,34,35,36]
IL-8	TAMs, T cells, ECs, HepSCs, CAFs, and cancer cells	Promotes cancer cell proliferation, angiogenesis, metastasis, an immunosuppressive TME, and chemoresistance	[37,38,39,40]
IL-10	M2 macrophages, Th2 cells, and Tregs	Promotes inflammation, cancer cell proliferation, cancer progression, and immunosuppression	[41,42]
IL-11	CAFs	Promotes inflammation and cancer cell proliferation	[43,44]
IL-12	M1 macrophages and neutrophils	Promotes inflammation	[45]
IL-13	Th2 cells and CAFs	Promotes inflammation, immunosuppression, and immune responses	[19]
IL-17A	Neutrophils, cancer cells, and CAFs	Promotes cancer cell proliferation and chemoresistance	[37,43]
IL-22	CD4+ T cells	Promotes cancer cell proliferation and chemoresistance	[37]
IL-33	TECs and cancer cells	Angiogenesis	[46,47,48]
MMPs	CAFs, TAMs, cancer cells, and HepSCs	TME remodeling	[29,34,49,50]
TNFα	M1 macrophages, TECs, KCs, and Th1 cells	Promotes inflammation, the EMT, migration, extravasion, angiogenesis, immune responses, immunosuppression, and M2 macrophage polarization	[24,25,28,32,51,52,53,54]
TGFβ	CAFs, M2 macrophages, Tregs, neutrophils, MDSCs, HepSCs, and cancer cells	Promotes the EMT, inflammation, immunosuppression, macrophage M2 polarization, fibroblast activation, and angiogenesis	[39,41,44,49,55,56,57,58]
VEGF	TECs, cancer cells, HepSCs, and CAFs	Promotes inflammation, immune responses, and angiogenesis	[38,59,60]
